# Curcumin Treatment Improves Motor Behavior in α-Synuclein Transgenic Mice

**DOI:** 10.1371/journal.pone.0128510

**Published:** 2015-06-02

**Authors:** Kateri J. Spinelli, Valerie R. Osterberg, Charles K. Meshul, Amala Soumyanath, Vivek K. Unni

**Affiliations:** 1 Jungers Center for Neurosciences Research, Oregon Health & Science University, Portland, Oregon, United States of America; 2 Department of Neurology, Oregon Health & Science University, Portland, Oregon, United States of America; 3 Research Services, Veterans Affairs Medical Center, Portland, Oregon, United States of America; 4 Department of Behavioral Neuroscience, Oregon Health & Science University, Portland, Oregon, United States of America; 5 Parkinson Center of Oregon, Oregon Health & Science University, Portland, Oregon, United States of America; Hertie Institute for Clinical Brain Research and German Center for Neurodegenerative Diseases, GERMANY

## Abstract

The curry spice curcumin plays a protective role in mouse models of neurodegenerative diseases, and can also directly modulate aggregation of α-synuclein protein *in vitro*, yet no studies have described the interaction of curcumin and α-synuclein in genetic synucleinopathy mouse models. Here we examined the effect of chronic and acute curcumin treatment in the Syn-GFP mouse line, which overexpresses wild-type human α-synuclein protein. We discovered that curcumin diet intervention significantly improved gait impairments and resulted in an increase in phosphorylated forms of α-synuclein at cortical presynaptic terminals. Acute curcumin treatment also caused an increase in phosphorylated α-synuclein in terminals, but had no direct effect on α-synuclein aggregation, as measured by *in vivo* multiphoton imaging and Proteinase-K digestion. Using LC-MS/MS, we detected ~5 ng/mL and ~12 ng/mL free curcumin in the plasma of chronic or acutely treated mice, with a glucuronidation rate of 94% and 97%, respectively. Despite the low plasma levels and extensive metabolism of curcumin, these results show that dietary curcumin intervention correlates with significant behavioral and molecular changes in a genetic synucleinopathy mouse model that mimics human disease.

## Introduction

Curcumin, the main component of turmeric spice, is a natural compound found in the root of the *Curcuma longa* plant. Of the three main curcuminoids found in the turmeric root, curcumin is the most abundant and the most biologically active. Much research has demonstrated that this polyphenolic compound has anti-oxidant, anti-inflammatory, and anti-amyloidogenic properties. These properties are particularly relevant for treatment of neurodegenerative diseases, and curcumin has been shown to alleviate cell death and neuronal loss in cellular and animal models of Alzheimer’s Disease (AD), Parkinson’s Disease (PD), Huntington’s Disease, and stroke [1,2]. Curcumin has been extensively studied in mouse models of AD, where it has been shown to reduce A-beta plaque size, reduce soluble A-beta, suppress soluble tau dimers, and lower oxidative stress [3–6]. While studies in mouse models of PD and Dementia with Lewy Bodies (DLB) are more limited, experiments from a toxin-induced mouse model of PD show that curcumin reverses neurodegeneration by blocking mitochondrial dysfunction and apoptosis [7].

In addition to promoting mitochondrial health and reducing oxidative stress, curcumin may be beneficial in PD and DLB by directly modulating aggregation of α-synuclein protein. α-synuclein is the most abundant protein found in Lewy Bodies (LBs), the pathological hallmark of PD and DLB, and is genetically and molecularly linked to neurodegeneration in both human patients and mouse models of disease [8,9]. Curcumin protects against α-synuclein-induced cell death in tissue culture cells by inhibiting mitochondrial toxicity and formation of reactive oxygen species [10,11]. Furthermore, curcumin directly binds to α-synuclein *in vitro*, and can both inhibit and reverse the formation of toxic α-synuclein aggregate species [12]. Molecular interaction studies reveal that curcumin binds to the non-amyloid-beta component (NAC) domain of α-synuclein to shield hydrophobic residues that drive self-aggregation, thus promoting and stabilizing non-aggregate forms of the protein *in vitro* [13]. Ahmad and colleagues also found that curcumin could dis-aggregate higher-order oligomeric and fibrillar forms of the protein, which are thought to be the toxic species. Because curcumin can dis-aggregate pre-formed α-synuclein aggregates, this suggests curcumin could potentially be used therapeutically, not just preventatively, to halt or reverse the disease process after it has already caused clinical symptoms.

To date, the effect of curcumin in genetic mouse models of PD, DLB, and related synucleinopathies that closely mimic human disease, has not been examined. We use transgenic mice with 2-3-fold over-expression of human GFP-tagged wild-type α-synuclein (Syn-GFP), which mimics duplication and triplication mutations found in some human patients [14,15]. Using a combination of *in vivo* multi-photon imaging through a cranial window and biochemical analysis of fresh and fixed brain tissue, we have previously characterized a pool of phosphorylated Syn-GFP microaggregates in cortical presynaptic terminals from these mice [15]. In this study, we examined the effect of both chronic and acute curcumin treatment on motor behavior, α-synuclein phosphorylation, and aggregation in these mice. We found that a moderate dose of dietary curcumin significantly improved the motor phenotype of Syn-GFP mice, and that this correlated with an increase in phosphorylated α-synuclein protein at cortical presynaptic terminals. We further found that acute treatment of curcumin for 2 weeks via i.p. injection did not significantly affect Syn-GFP aggregates *in vivo* or in fixed and fresh brain tissue, but did have the same effect of increasing phosphorylated α-synuclein. Combined these results indicate that curcumin intervention promotes phosphorylation of α-synuclein, which may contribute to improved motor behavior over time.

## Experimental Procedures

### Mice

Mice were housed by Oregon Health and Science University’s Department of Comparative Medicine (DCM) or the Veterans Affairs Medical Center’s Veterinary Medical Unit, held in a light-dark cycle, temperature- and humidity-controlled animal vivarium, and maintained under ad libitum food and water diet supplied by the DCM. Syn-GFP (PDNG78 line; [14]) heterozygous male mice were mated to BDF1 females from Charles River Laboratories. Male and female mice between 3 and 10 months old were used for experiments. All experiments were approved by the OHSU Institutional Animal Care and Use Committee (Protocol number: IS01185) and every effort was made to minimize the number of animals used and their suffering.

#### Chronic and acute curcumin treatments

Mouse diet containing 500 ppm dietary supplement grade curcumin (Chromadex, Irvine, CA), premixed in soybean oil prior to incorporation into the diet, or control diet containing an equal amount of oil without curcumin, was made by Harlan Teklad. For chronic treatment, age matched male and female mice (n = 2 per sex per treatment group) were placed on the modified diet at 3 months of age, and were maintained on the diet for 6 months until the time of sacrifice. Mice were single housed, and animals and diet were weighed every day for 5 days at the start of the diet intervention, then weekly thereafter to measure weight gain and consumption. For acute curcumin treatment, mice with previously installed cranial windows were treated with 15 mg/kg/day dietary supplement grade curcumin or 50% DMSO/D-PBS vehicle control i.p. daily for 15 days. Mice were given soft food and daily i.p. injections of D-PBS to maintain hydration for the duration of the treatment. Imaging experiments were performed one day prior to the start of treatment, and at 7 and 14 days of treatment. On day 15 of treatment, mice were sacrificed 5 hours after the last curcumin or DMSO injection.

### Behavioral Analysis

1 day prior to behavioral testing, mice were acclimated to the testing room for 30 minutes. Gait analysis was performed on a DigiGait treadmill (Mouse Specifics, Quincy, MA), with a speed of 24 cm/sec, with 2–4 seconds of video analyzed for each mouse. In between animals, the treadmill was wiped down with Nolvasan and 70% ethanol. Mice were run on a declined platform (achieved by elevating the back end of the treadmill 8 inches off the ground) on day 1 of testing, rested for 4 days, then run on a flat platform. Muscle strength was tested using a Grip Strength Meter (Columbus Instruments, Columbus, OH); all limbs, then forelimbs only were tested.

#### Tissue Collection

Mice were anesthetized with ketamine/xylazine (100mg/kg/10mg/kg), and blood was collected from the heart into heparinized tubes using the cardiac puncture technique. Plasma was isolated by centrifugation at 2,000 x g for 10 minutes, and plasma samples were immediately snap frozen in an ethanol/dry ice bath. Following blood removal, animals were sacrificed via cervical dislocation, and the brain was dissected out of the skull, cerebellum and olfactory bulbs were removed, and the remaining tissue was bisected along the midline. Half the brain was fixed in 4% paraformaldehyde in a circulating water bath for 1 h, 150 W, at 30°C using a BioWave (Pelco, Ted Pella, Inc, Redding, CA) microwave fixation system, then postfixed in 4% paraformaldehyde overnight at 4°C. The other half of the brain was homogenized in Syn-PER Synaptic Protein Extraction Reagent (Thermo Fisher), and synaptosome and cytosolic components were isolated according to the manufacturers protocol. Fixed brain tissue was mounted on a Leica VT1000 S Vibratome and sagittally sectioned into 50 μm slices, which were then stored in PBS/0.05% sodium azide at 4°C.

### LC-MS/MS Analysis

LC-MS/MS analysis was based on a previously validated method for quantitation of curcumin in plasma [16]. Experimental plasma samples and blank mouse plasma spiked with known amounts of curcumin were treated as follows, in triplicate. Frozen samples were thawed on ice, and 50 μL of sample was mixed with 150 μL acetonitrile containing 0.15 ng/μL honokiol internal standard (Sigma). For enzyme treatment, curcumin spiked plasma standards and experimental plasma samples (50 μL) were incubated with 5 μL B-glucuronidase (20 units, Type H-2, Sigma G0876) at 37°C for 1 hour. Precipitated protein was separated by centrifugation at 8,000 x g for 5 minutes. The remaining supernatant was filtered through 0.22 μ Millipore Durapor PVDF spin filter, and 10 μL was injected onto a Zorbax Extend-C18 column (4.6x150mm, 5 μm, Agilent, Palo Alto, CA). The mobile phase was a mixture of (A) water and (B) acetonitrile, both containing 0.02% formic acid. The flow rate was 0.8 mL/min, and the following gradient was applied: 50–95% B over 0–5 min, 95% B for 3 min, followed by a return to 50% B and equilibration. For MS/MS analysis the Shimadzu Prominence HPLC was interfaced to an Applied BioSystems 4000 Q-TRAP hybrid triple-quadrupole, linear ion trap mass spectrometer with electrospray interface (ESI) used in negative ion mode, with the following source parameters: source voltage, -4.5 kV; source temperature, 500°C; Ion source gas 1, 20; ion source gas 2, 50l/h; curtain gas, 15; interface heater, on. Curcumin and honokiol were measured using multiple reaction monitoring (MRM) and optimal parameters were determined by direct infusion of each compound (Table 1). The collision gas was set to medium.

**Table 1:** LC-MS/MS parameters.

Q1 mass	Q3 mass	Time (msec)	ID	DP	EP	CE	CXP
265.0	224.1	150	honokiol	-100	-10	-32	-7
265.0	222.6	150	honokiol	-100	-10	-42	-15
367.0	133.6	150	curcumin	-85	-10	-46	-11
367.0	149	150	curcumin	-85	-10	-24	-9
367.0	217	150	curcumin	-85	-10	-15	-10

### Immunohistochemistry

Human frontal cortex from a patient with Dementia with Lewy Bodies (DLB) and a normal healthy control were obtained from the OHSU Brain Bank, and 50 μm sections were cut on a Leica VT1000 S Vibratome. To detect phosphorylated α-synuclein in both human and mouse tissue, brain sections were blocked in 0.1% Triton-X, 10% goat serum for 1–2 hours, then stained with rabbit monoclonal S129-P-α-synuclein antibody (Abcam, catalogue number ab51253, 1:1000 for mouse tissue, 1:500 for human tissue) overnight at 4°C, washed extensively, then stained with goat anti-rabbit Alexa-647 (Invitrogen) at 1:2000 overnight at 4°C. Mouse brain tissue was mounted in CFMR2 (Citifluor, London, UK), which partially cleared the tissue to allow for deeper imaging depths, and imaged on a Zeiss LSM 780 confocal microscope. IMARIS software (Bitplane, Zurich, Switzerland) was used to quantify GFP and S129-P fluorescence at individual synapses, and the CoLoc function was used to analyze S129-P/GFP colocalization. For curcumin or Thioflavin-S (ThS) co-staining in human brain tissue, sections were first stained with antibodies, then washed in 70% ethanol, incubated with 10 μM curcumin or 0.05% ThS in 50% ethanol for 10–15 minutes, washed again in 70% ethanol, and rehydrated in water. Tissue was mounted in Vectashield (Vector Labs, Burlingame, CA) and imaged on a Zeiss LSM 780 confocal microscope equipped with the spectral imaging with linear unmixing module. Spectral imaging was performed in lambda mode using a 9.7nm gradient width and 34 PMT detectors for a 419–691 nm wavelength range.

### Proteinase-K and Western Blot analysis

Proteinase-K-resistant aggregates were analyzed by incubating synaptosome and cytosolic brain fractions with 10 μg/mL Proteinase-K (Thermo Scientific) for 30 minutes at 37°C, followed by SDS-PAGE western blot using the Li-cor Odyssey CLx quantitative western blot imaging system. Syn-GFP blot signal was detected using a rabbit polyclonal antibody against GFP (Abcam, 1:2000), and data was quantified using LiCor Image Studio software.

### Cranial window surgery and *in vivo* imaging

Cranial window surgery, *in vivo* imaging and photobleaching experiments were performed as previously described [15]. Briefly, mice were anesthetized with isoflurane (1–2%) and a cranial window was surgically installed on the skull. Following surgery, animals were monitored for pain and given daily i.p. injections of buprenorphine for 1–3 days, as needed. Imaging experiments were begun at least 3 weeks following placement of the cranial window. For multiphoton imaging, animals were anesthetized with isoflurane, mounted to a custom stereotaxic frame, and imaged using a 20x/1.0NA water immersion objection and a Zeiss LSM 7MP multiphoton microscope, outfitted with dual channel BiG (binary GaAsP) detectors and the Coherent Chameleon titanium-sapphire femtosecond pulsed laser source, tuned to 860 nm. Baseline images of Syn-GFP-labeled cell bodies and presynaptic terminals in cortical layers 2/3 were obtained using 1–3 mW power (measured at objective exit). Large square regions of interest (ROI, 53x53 μm^2^) encompassing 50–100 terminals were bleached with ~100–150 mW power over <10 ms, and recovery images were acquired at 1–5 minute intervals, for 15 minutes. Zeiss Zen 2011 image acquisition software was used for image acquisition, and data was analyzed using Fiji image analysis software (http://fiji.sc/). The background-subtracted fluorescence intensity of the ROI was calculated for each time point and graphed as normalized fluorescence intensity vs. time using Prism software (GraphPad, La Jolla, CA). The data was fit as a one-phase association to calculate the tau recovery rate and the immobile fraction.

### Statistics

All statistical analyses were carried out using Prism software (Graphpad, La Jolla, CA), and all data are presented as mean ± standard deviation. For behavioral analysis, right and left paw data was averaged for the fore paws or hind paws for each animal, then data for each animal in a group were averaged, to yield an n = 4 animals per group. Unless otherwise noted, unpaired, two-tailed t-tests were used to determine significance between groups. Since no significant differences were detected between male and female mice, results from both sexes were combined.

## Results

### Curcumin binds to Lewy Bodies in diseased human brain tissue

To eliminate confounding effects due to variations in purification methods or the presence of other curcuminoids, we used synthetic curcumin, as opposed to turmeric root or extracted curcumin. We found that curcumin from Chromadex was >99% pure by HPLC, stable in 100% DMSO solution over 1 week, and stable as a powder for over 1 year.

**Fig 1:**
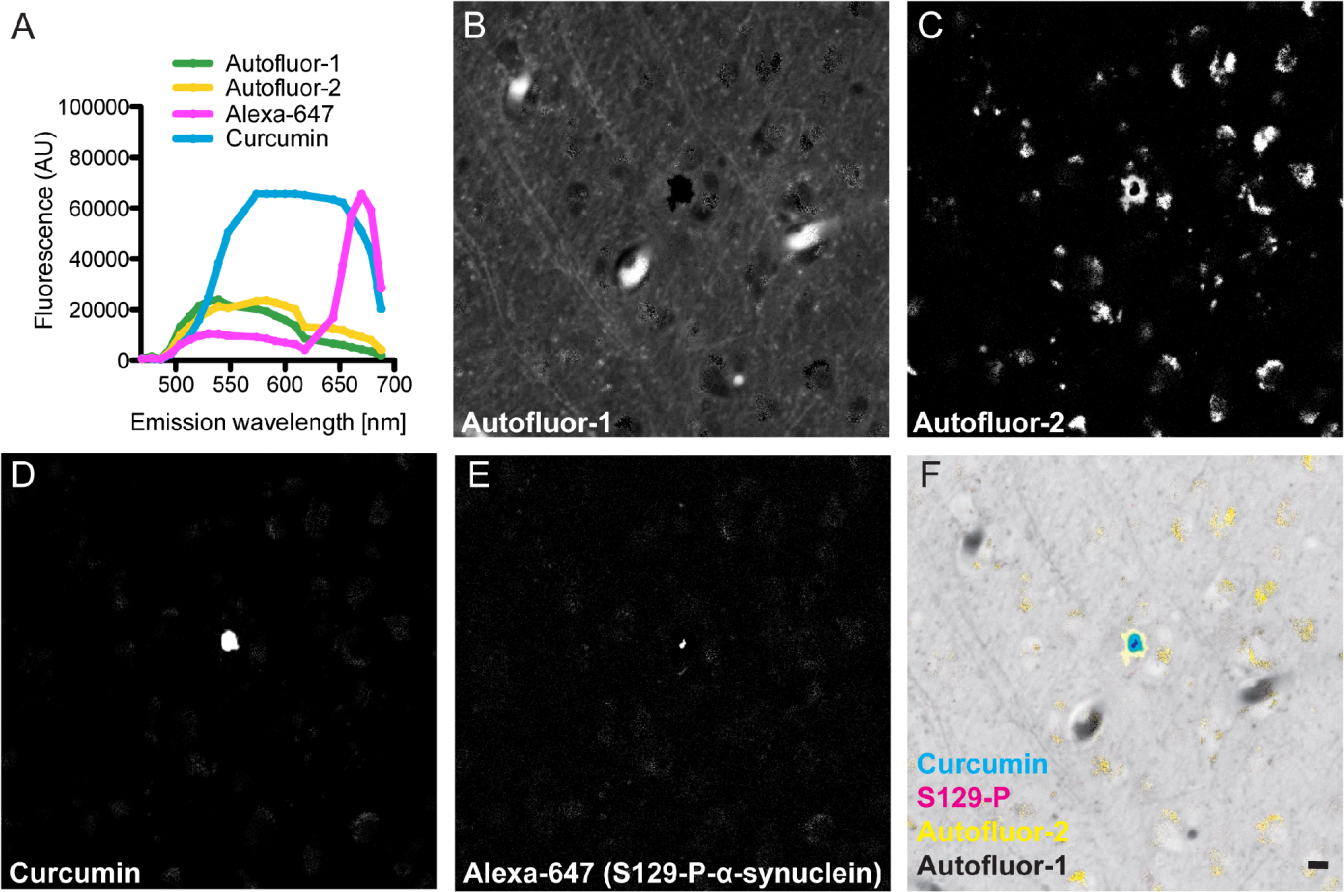
Curcumin stains Lewy Bodies. (A) Individual fluorescence spectra of curcumin, Alexa-647 secondary antibody, and two autofluorescent components, defined by spectral imaging and linear unmixing. Autofluorescence components (B, C) were clearly separated from adjacent curcumin-positive (D) structures, which were defined as Lewy Bodies because they co-labeled with S129-P-α-synuclein-A647 antibodies (E, G). Minimal unassigned residual light (F) demonstrates a high efficiency of the unmixing technique for the defined spectra. (Scale bar = 10 μm)

We next characterized the ability of curcumin to bind to LBs in diseased human brain tissue. Previous data has shown that curcumin labels human A-beta plaques and neurofibrillary tangles, suggesting a specific preference for proteins in a beta-sheet rich amyloid confirmation, similar to dyes such as Thioflavin-S/T and Congo Red [5,17,18]. Because human brain tissue contains significant amounts of lipofuscin autofluorescence, which overlaps with the broad curcumin emission spectrum, we found that spectral imaging combined with linear unmixing was useful to accurately detect curcumin staining above the autofluorescent background in the tissue. Using a Zeiss 780 laser scanning confocal microscope with spectral unmixing capabilities, we were able to isolate two autofluorescent spectra, as well as separate spectra for curcumin and Alexa-647 secondary antibodies, with minimal unassigned residual light (Fig 1A). This technique resulted in clear separation of each fluorescent component, particularly the autofluorescence-2 channel and curcumin, which have closely overlapping spectra that are difficult to tease apart using regularly available emission filters [5]. Using this technique, we found that curcumin binds to Serine-129-phosphorylated (S129-P)-α-synuclein-positive cortical LBs in human brain tissue from patients with DLB (Fig 1B–1G). While it appeared as though the LBs may contain a smaller central core of S129-P-α-synuclein, with a larger region of curcumin staining outside this core, we believe that this is likely a result of the software attempting to clearly assign only one spectral component to the LB core. In normal control brain tissue, no staining for S129-P-α-synuclein was seen (S1 Fig). To our knowledge, this is the first report of curcumin binding to LBs in human brain tissue.

### Free and glucuronidated curcumin are present in plasma from mice on a curcumin diet

**Fig 2:**
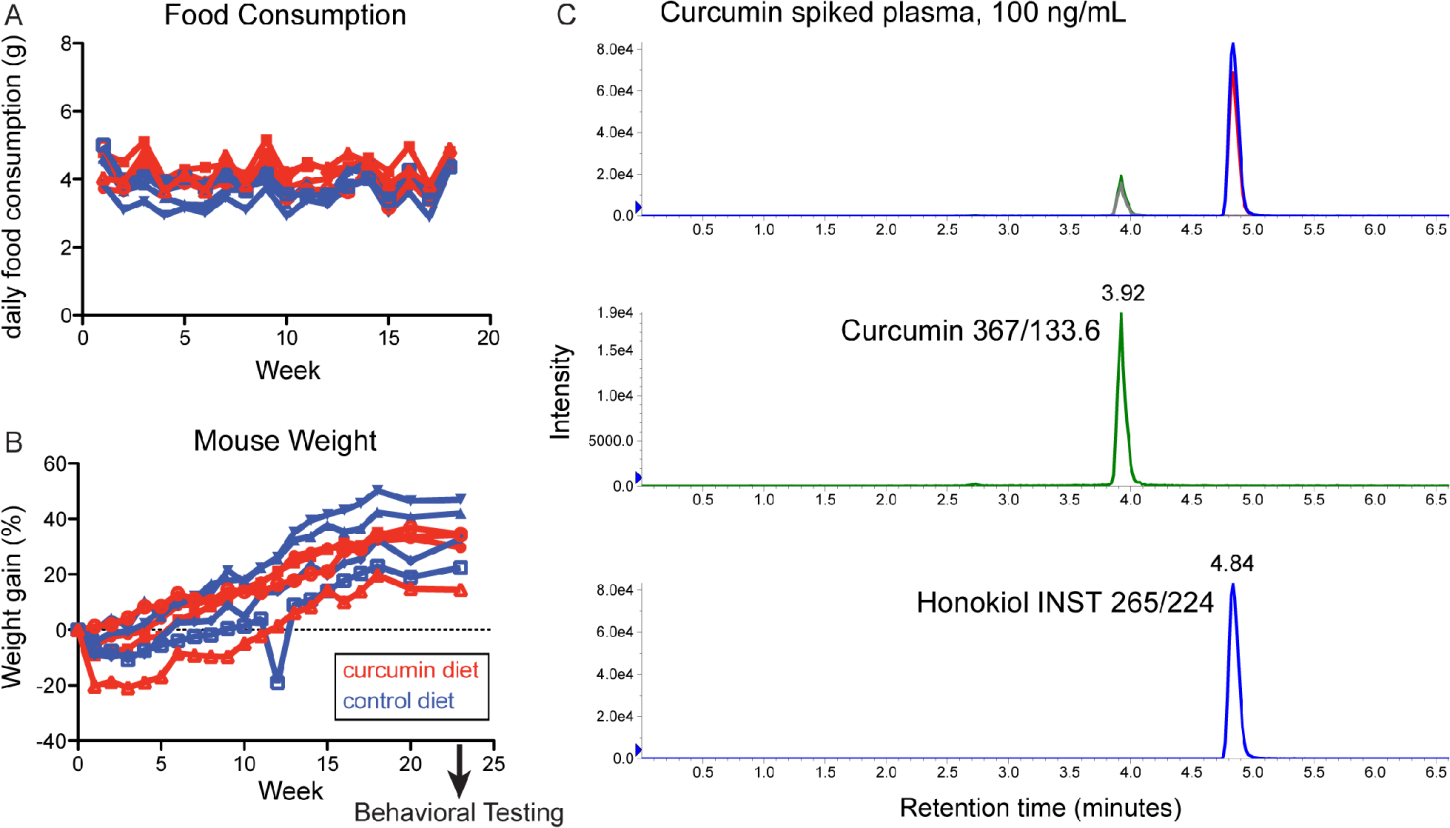
LC-MS/MS detection of curcumin in plasma from mice on a curcumin diet. Food consumption (A) and body weight (B) for mice on a 500 ppm curcumin diet or control diet, recorded weekly for 6 months. Extracted-ion chromatogram for plasma matrix spiked with 100 ng/mL curcumin (C), showing distinct peaks for curcumin and honokiol internal standards.

Given this data that curcumin can bind to, and therefore potentially modulate, aggregated α-synuclein in human tissue, we next set out to test its effects in synucleinopathy mouse models. To examine the long-term effects of curcumin, we treated Syn-GFP mice with 500 ppm curcumin diet or control diet for 6 months. Food consumption and body weight were monitored weekly; no significant differences between curcumin and control diet mice were found at any time point (Fig 2A and 2B). Motor behavior was assessed at 23 weeks, and brain tissue and plasma was harvested at 24 weeks.

We used a previously validated liquid chromatography tandem mass spectrometry (LC–MS/MS) method to measure curcumin levels in plasma samples after dietary treatment [16]. In both spiked control plasma matrix and experimental samples, we detected a single peak corresponding to curcumin (molecular weight 368) by following the 367/133 and 367/217 MRM transitions (Fig 2C). The 367/133 transition values were used for quantification of curcumin. The lower limit of quantitation (LLOQ) was calculated as 5 ng/mL due to this being the lowest value on the standard curve, although curcumin peaks were clearly detectable with signal to noise ratios greater than 10:1 at lower concentrations, and the lower limit of detection (LLOD) was 0.033 ng/mL. Standard curves were linear in the range of 0–500 ng/mL curcumin (r = 0.998±0.002). We found an average plasma curcumin concentration of < 5 ng/mL (4.40±5.72 ng/mL) in 3 out of 4 mice on a curcumin diet (Table 2). One sample did not contain detectable curcumin, likely because of rapid metabolism of free curcumin (see below) and a delayed period of time between the last food consumption time point (at the end of the dark cycle in the early morning) and sacrifice (early afternoon). We did not detect curcumin in plasma from control diet mice.

**Table 2:** Levels of curcumin in plasma from chronic and acutely treated mice.

Mouse ID	Treatment	Free Curcumin (ng/ml)	Free + Glucuronidated Curcumin (ng/mL)	% Glucuronidated Curcumin
				
#179—curcumin	chronic	1.0 ± 0.08	27.33 ± 1.92	96.3
#164—curcumin	chronic	11.0 ± 1.03	2.22 ± 0.13	NA
#165—curcumin	chronic	1.2 ± 0.27	8.47 ± 0.73	85.9
#183—curcumin	chronic	ND	12.05 ± 0.21	100.0
#210—curcumin	acute	8.66 ± 0.62	481.00 ± 20.95	98.2
#299—curcumin	acute	13.07 ± 0.21	364.67 ± 52.29	96.4
#192—curcumin	acute	12.93 ± 0.51	273.00 ± 7.81	95.3

For mice treated chronically with a curcumin diet for 6 months, average free curcumin in plasma was 4.40±5.72 ng/mL and average free plus glucuronidated curcumin was 12.52±10.68 ng/mL, indicating that 94.1±7.3% of curcumin was glucuronidated. For mice treated acutely with 15 mg/kg/day curcumin i.p. for 15 days and sacrificed 5 hours after the final treatment, average free curcumin was 11.55±2.51 ng/mL and average free plus glucuronidated curcumin was 372.89±104.24 ng/mL, indicating that 96.6±1.5% of curcumin was glucuronidated (samples from individual mice were run in triplicate; mean ± SD; ND, not detectable; NA, not applicable).

Curcumin is rapidly metabolized *in vivo* through two pathways—reduction of the compound to dihydrocurcumin, tetrahydro-curcumin, and hexahydrocurcumin, and addition of a sugar group to yield curcumin-glucuronide versions of the parent compound and each of the three metabolites. Previous research has shown that up to 99% of curcumin is rapidly glucuronidated *in vivo* [19]. To examine the levels of curcumin-glucuronide in mice on a curcumin diet, we treated plasma samples with B-glucuronidase prior to LC-MS/MS, to remove the sugar and reduce the compound back to free curcumin. After enzyme treatment, we found an average curcumin concentration of 12.52±10.68 ng/mL, indicating that 94.1±7.3% of curcumin was glucuronidated in plasma from curcumin diet mice (Table 2). Enzyme treatment did allow us to detect curcumin in the mouse sample that had no free curcumin (mouse #183), indicating that 100% of the curcumin in this mouse was glucuronidated. Surprisingly, mouse #164 had a lower concentration of curcumin in plasma after enzyme treatment, perhaps indicating that the curcumin in this sample had degraded during freeze/thaw cycles between the initial mass spec run and the enzyme treatment experiment.

### Curcumin diet intervention improves motor behavior

**Fig 3:**
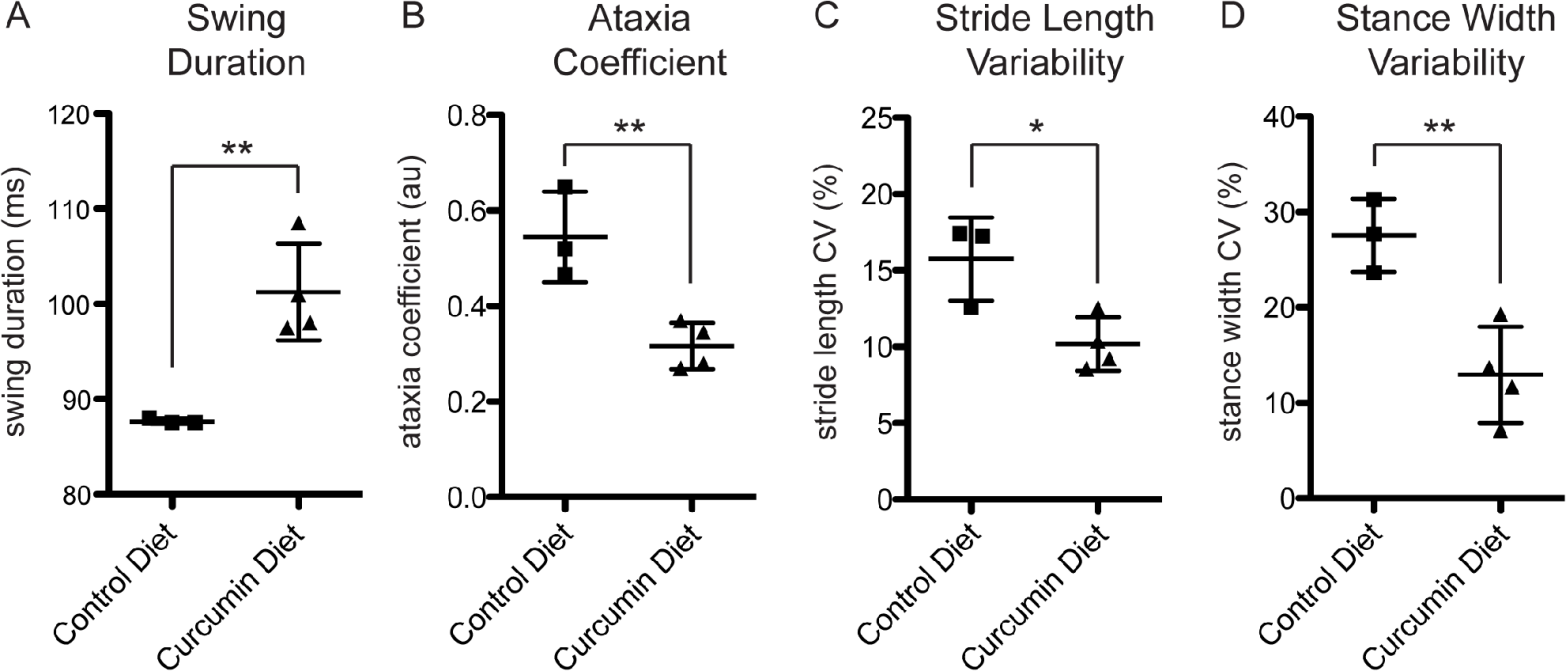
Curcumin diet mice show improved gait. Syn-GFP mice on a 6 month curcumin diet intervention had significant changes in gait compare to control diet mice, including increased fore paw swing duration (A), decreased fore paw ataxia coefficient (B), decreased for paw stride length variability (C), and decreased fore paw stance width variability (D). The ataxia coefficient, and variability in stride length and stance width, are all associated with a PD-like phenotype in both humans and mice (flat platform run, n = 4 animals per group, *p<0.05, **p<0.01).

Automated gait analysis is a highly sensitive tool for measuring changes in multiple gait parameters in mouse models of PD, Huntington’s Disease, and Amyotrophic Lateral Sclerosis [20]. To examine the effects of curcumin intervention on motor behavior, we tested curcumin and control diet mice using automated gait analysis on the DigiGait machine, and measured changes in several parameters. On a declined platform, there was a significant increase in hind paw angle in curcumin diet mice (5.0±2.5 degrees for control diet, 14.1±5.6 degrees for curcumin diet, p<0.05). On a flat platform, mice on a curcumin diet showed a significant increase in fore paw swing duration (87.7±0.3 ms for control diet, 101.3±5.1 ms for curcumin diet, p<0.01; Fig 3A) and a non-significant trend towards a decrease in hind paw stride frequency (3.9±0.1 steps/sec for control diet, 3.6±0.2 steps/sec for curcumin diet, p = 0.0671), compared to control diet mice. Combined, these two parameters indicate that control diet mice took a greater number of shorter steps in a given time period, which is consistent with gait changes reported in a toxin-induced mouse model of PD [21]. These altered gait dynamics in our mouse model are due to expression of the Syn-GFP transgene, and curcumin diet intervention was able to partially normalize this deficit.

Strikingly, the ataxia coefficient, an index of step-to-step variability that is increased in human PD patients, was significantly decreased in the fore paws for curcumin diet mice compared to control (0.55±0.10 for control diet, 0.32±0.05 for curcumin diet, p<0.01; Fig 3B). Two other PD-associated parameters, the variability in stride length and stance width in the fore paws, were also decreased in curcumin diet mice (stride length variability = 15.76±2.72% for control diet, 10.18±1.74% for curcumin diet, p<0.05; stance width variability = 27.55±3.85% for control diet, 12.95±5.05% for curcumin diet, p<0.01; Fig 3C and 3D). Stride length variability is higher in patients with PD [22,23]. In mice, the normal range is 10–12% and is increased in a toxin-induced mouse model of PD [21]. Increased stance width variability is associated with PD in human patients, and is normally in the range of 10–15% in mice [21,24]. While control diet mice fall outside of the normal range for both of these parameters, curcumin diet intervention normalized the effect of over-expressing the Syn-GFP transgene to return these mice to the normal range.

We also tested muscle strength in curcumin and control diet mice, using a Grip Strength Meter, and found no significant difference between groups (control diet = 2.65±0.31 Newtons, curcumin diet = 2.62±0.29 Newtons; p = 0.8723). This result is consistent with a separate study that showed no change in grip strength in a Pink1 genetic model of PD [25]. This supports the idea that the motor phenotype we detect is similar to PD-like phenotypes, and is not a result of changes in muscle strength.

### Curcumin diet intervention results in an increase in phosphorylated α-synuclein

**Fig 4:**
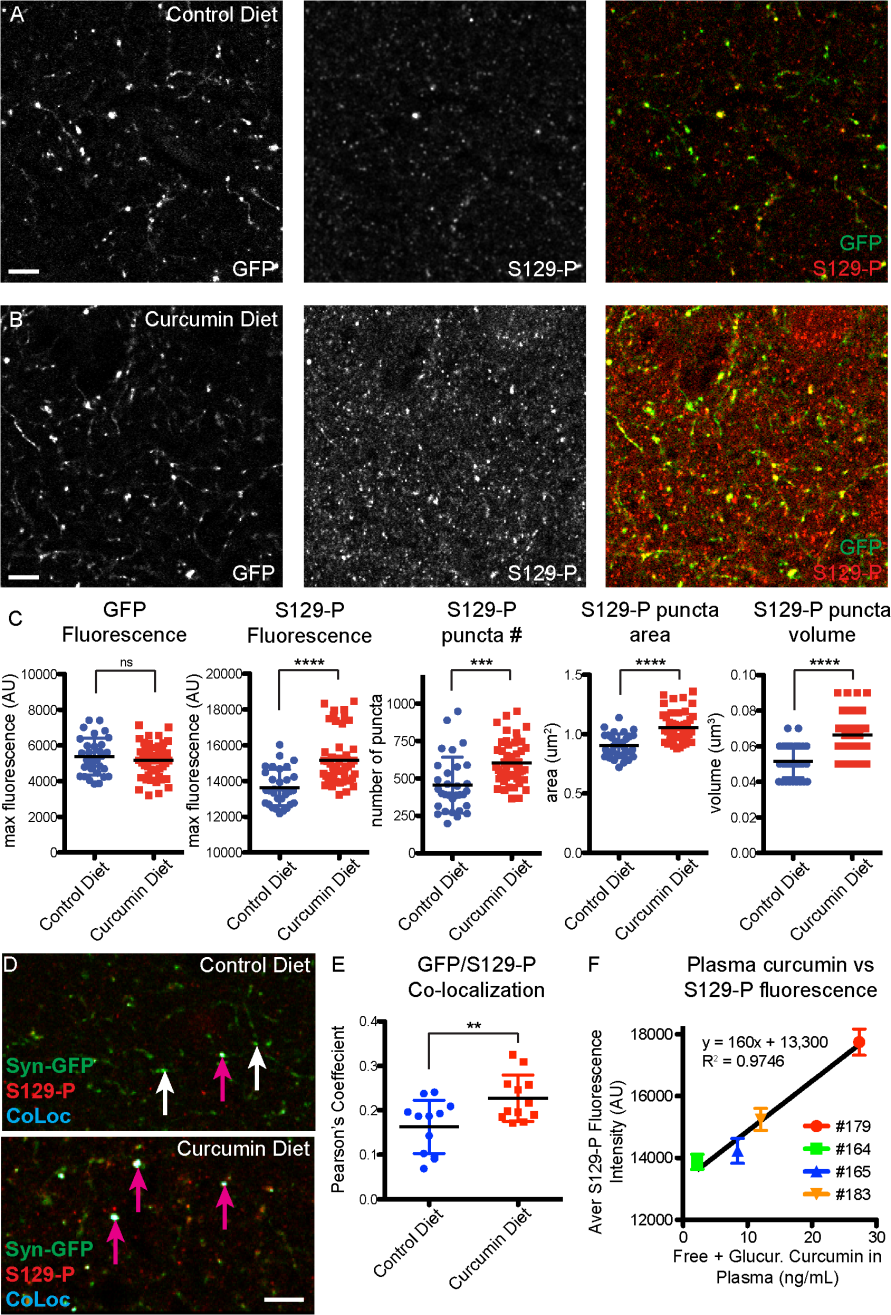
Increased S129-P-α-synuclein in curcumin diet mice. Immunohistochemical detection of S129-P-α-synuclein in cortical tissue from control (A) and curcumin (B) diet mice. (C) Quantification of GFP and S129-P fluorescence at individual presynaptic terminals shows significant changes in S129-P puncta, but no change in GFP. (D) Analysis of GFP/S129-P colocalization (CoLoc channel), and quantification of the Pearson’s Colocalization Coefficient (E), demonstrates an increase in S129-P at GFP-positive terminals. (F) Strong correlation between plasma curcumin levels and terminal S129-P fluorescence in individual mice. (n = 4 mice for control diet, n = 4 mice for curcumin diet; each data point represents the average value for a 3μm z-stack, with 7–15 z-stacks analyzed per mouse; pink arrows indicate colocalized synapses, white arrows indicate lack of colocalization; **p<0.01, ***p<0.0005, ****p<0.0001, Scale bar = 5 μm).

We next examined molecular changes in α-synuclein as a result of curcumin diet intervention, using immunohistochemical and biochemical assays on fixed and fresh brain tissue. We previously found that presynaptic Syn-GFP terminal microaggregates contain S129-P forms of α-synuclein, and are resistant to digestion by Proteinase-K [15]. Here we find that staining for S129-P-α-synuclein was increased in presynaptic terminals in mice that were on a curcumin diet (Fig 4A and 4B). We found that there was no change in GFP fluorescence in presynaptic terminals, but there was an increase in the number, area, volume, and fluorescence of presynaptic S129-P puncta (control diet GFP fluorescence = 5373±1039 AU, curcumin diet GFP fluorescence = 5171±955 AU, p = 0.3767; control diet S129-P fluorescence = 13641±1006 AU, curcumin diet S129-P fluorescence = 15170±1471 AU, p<0.0001; control diet S129-P number = 454±186, curcumin diet S129-P number = 604±146; p<0.0005; control diet S129-P area = 0.9±0.1 μm^2^, curcumin diet S129-P area = 1.1±0.1 μm^2^, p<0.0001; control diet S129-P volume = 0.052±0.009 μm^3^, curcumin diet S129-P volume = 0.066±0.011 μm^3^, p<0.0001; Fig 4C). When we looked exclusively at Syn-GFP-positive terminals, we also detected a significant increase in S129-P levels, as measured by analyzing the amount of colocalization between S129-P and Syn-GFP (control diet Pearson’s coefficient = 0.16±0.06, curcumin diet Pearson’s coefficient = 0.23±0.5, p<0.01, Fig 4D and 4E). Combined, these IHC findings indicate that chronic exposure to curcumin results in a global increase in α-synuclein phosphorylation state, at both synapses that contain endogenous mouse α-synuclein and synapses that over-express the human Syn-GFP protein. We also found a strong correlation between plasma levels of curcumin and S129-P fluorescence intensity, in individual mice (Fig 4F).

**Fig 5:**
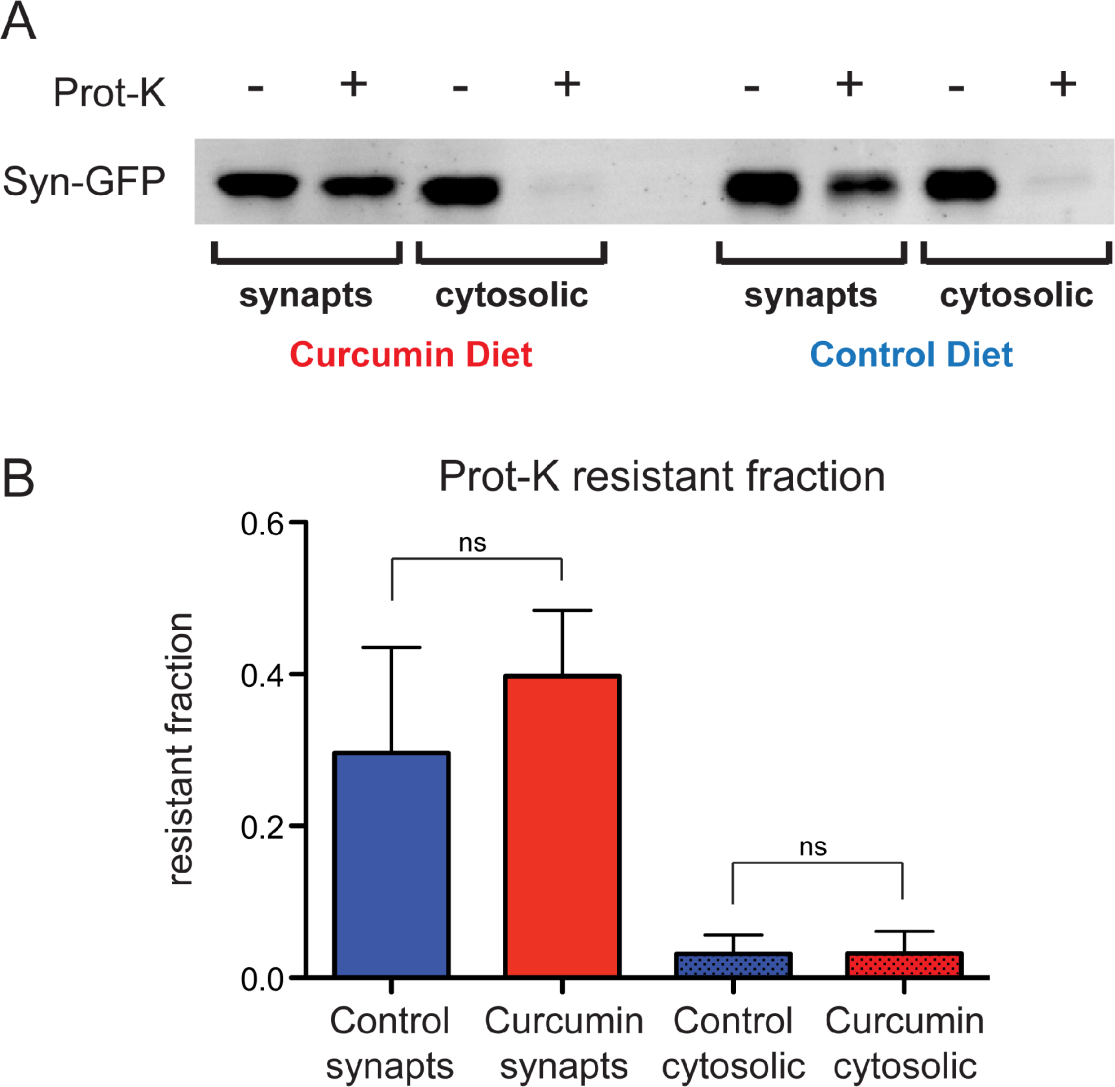
No change in Proteinase-K-resistant aggregates in curcumin diet mice. (A) Western blot detection of Syn-GFP microaggregates, following Proteinase-K digestion of synaptosome and cytosolic protein fractions from control and curcumin diet mice. (B) Quantification of Syn-GFP band intensity shows no change in the resistant fraction between control and curcumin diet mice.

To biochemically examine aggregates, we isolated synaptosome and cytosolic proteins from whole brain extracts of mice that were on a curcumin or control diet. Our previous work demonstrated that Syn-GFP mice contain a single species of Syn-GFP, identified by molecular weight under denaturing SDS-PAGE western blot conditions, and do not contain multiple SDS-resistant aggregate species of different molecular weights [15]. We further found that ~35% of Syn-GFP in synaptosomes is resistant to digestion by Proteinase-K, compared to ~2% of cytosolic Syn-GFP [15]. In curcumin diet mice, we did not detect any significant changes in the Syn-GFP Proteinase-K-resistant fraction in synaptosomes or cytosolic compartments, indicating that chronic curcumin treatment has no effect on Proteinase-K-resistant aggregates in these mice (control diet synaptosome fraction = 0.30±0.14, curcumin diet synaptosomes fraction = 0.40±0.09, p = 0.2654; control diet cytosolic fraction = 0.03±0.02, curcumin diet cytosolic fraction = 0.03±0.03, p = 0.9762; Fig 5).

### Acute curcumin treatment modulates phosphorylation of α-synuclein, but has no effect on *in vivo* aggregation

**Fig 6:**
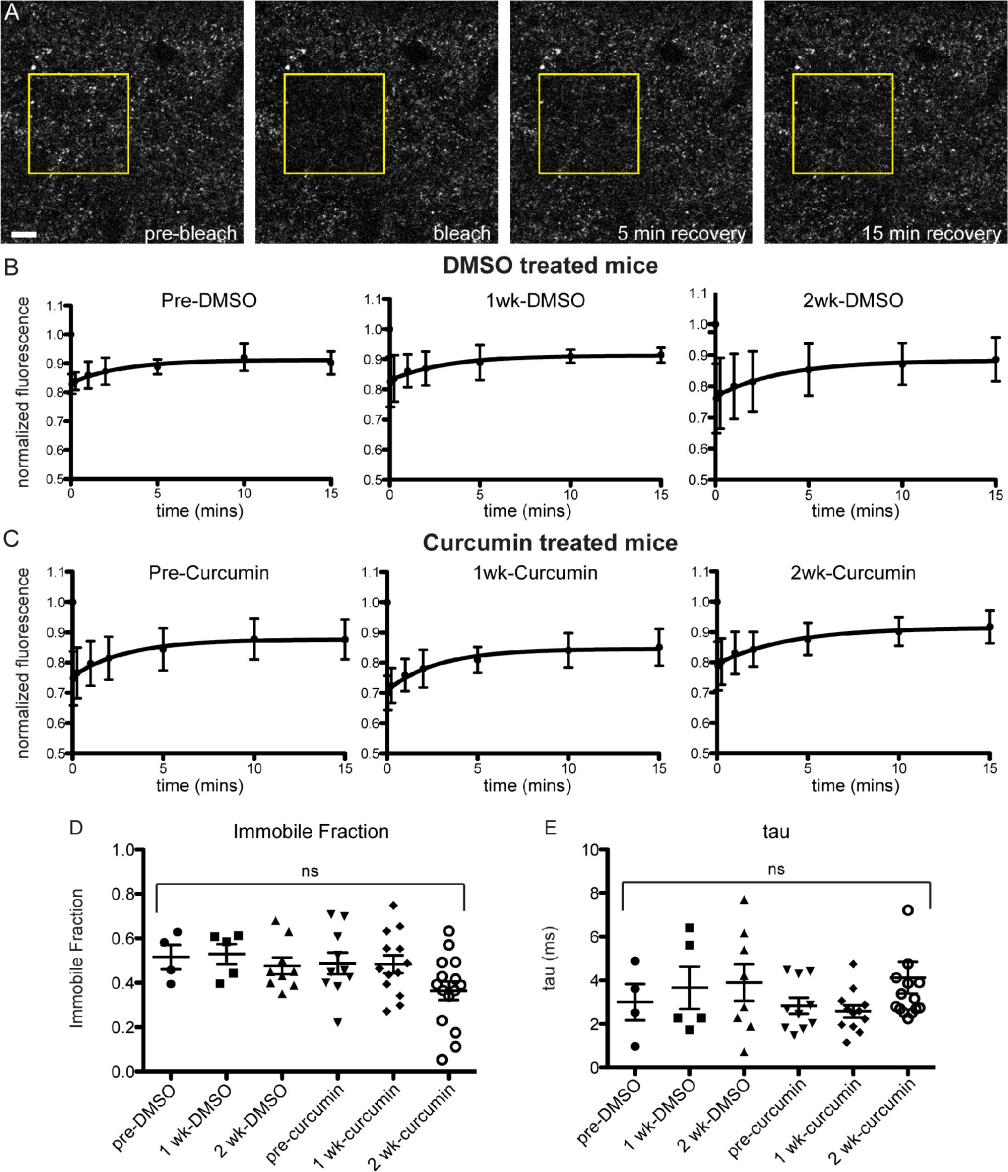
No change in in vivo FRAP kinetics in acutely treated curcumin mice. (A) Photobleaching and recovery imaging of presynaptic Syn-GFP protein in cortical brain tissue, through a cranial window in the skull. (B) FRAP recovery curves from DMSO and curcumin (C) treated animals, at pre-treatment, 1 week, and 2 week time points. (D) Immobile Fraction and recovery time constant tau values (E) do not change over time or with treatment (n = 3 animals curcumin, n = 2 animals DMSO, 2–5 regions per animal per time point; yellow box indicates bleach ROI; Scale bar = 10 μm).

To look more directly at the effect of curcumin on terminal microaggregates *in vivo*, we adopted an acute curcumin treatment paradigm that we could apply to Syn-GFP mice that had surgically-installed cranial windows over the cortex. We have shown previously that Syn-GFP terminal microaggregates have slower recovery kinetics and a significant immobile fraction in Fluorescence Recovery After Photobleaching (FRAP) experiments, compared to soluble Syn-GFP in the cell body [15]. We treated mice with 15 mg/kg/day curcumin or DMSO vehicle control i.p. for 15 days, and examined FRAP kinetics at pre-injection, 7 day, and 14 day time points. We found no significant change in the immobile fraction (IF) or the tau recovery rate due to curcumin treatment (pretreatment DMSO IF = 0.52±0.11, 1 week DMSO IF = 0.53±0.10, 2 week DMSO IF = 0.48±0.11, pretreatment curcumin IF = 0.49±0.15, 1 week curcumin IF = 0.48±0.14, 2 week curcumin IF = 0.36±0.16; pretreatment DMSO tau = 3.00±1.66 ms, 1 week DMSO tau = 3.66±2.17 ms, 2 week DMSO tau = 3.90±2.38 ms, pretreatment curcumin tau = 2.83±1.16 ms, 1 week curcumin tau = 2.58±0.96 ms, 2 week curcumin tau = 2.56±5.68 ms; Fig 6). A 2-way repeated measures ANOVA showed that there no effect of time or treatment, and no interaction effect (interaction p = 0.6927, time p = 0.1846, treatment p = 0.1471), although there was a trend towards a decrease in the immobile fraction over time in some curcumin regions analyzed.

**Fig 7:**
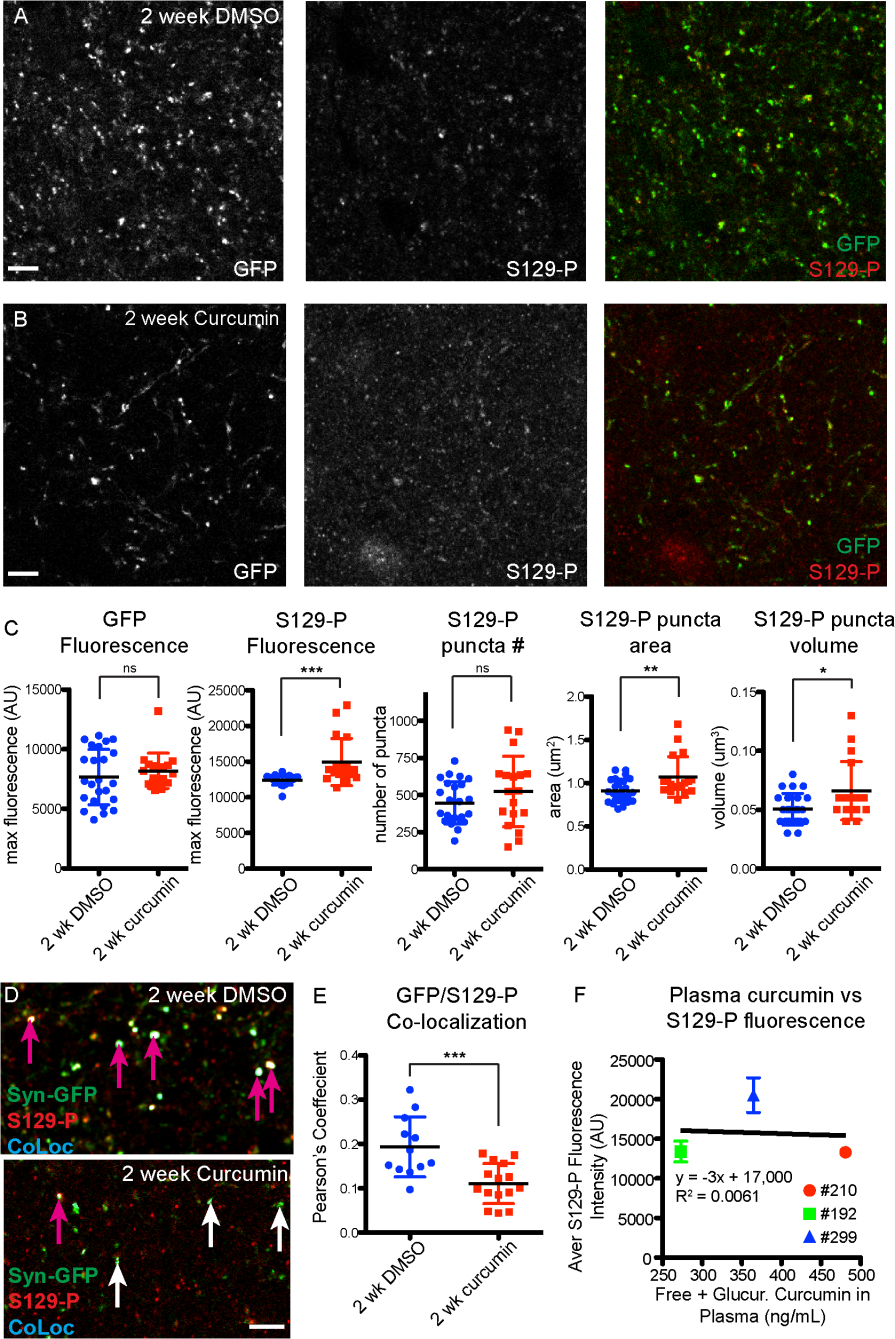
Increased S129-P-α-synuclein in acutely treated curcumin mice. Immunohistochemical detection of S129-P-α-synuclein in cortical tissue from mice treated for 2 weeks with DMSO (A) or 15mg/kg/day curcumin (B). (C) Quantification of GFP and S129-P fluorescence at individual presynaptic terminals shows significant changes in S129-P puncta, but no change in GFP. (D) Analysis of GFP/S129-P colocalization (CoLoc channel), and quantification of the Pearson’s Colocalization Coefficient (E), shows a decrease in S129-P at GFP-positive terminals. (F) No correlation between plasma curcumin levels and terminal S129-P fluorescence in individual mice. (n = 2 mice for DMSO treatment, n = 3 mice for curcumin treatment; each data point represents the average value for a 3μm z-stack, with 4–12 z-stacks analyzed per mouse; pink arrows indicate colocalized synapses, white arrows indicate lack of colocalization; *p<0.05,**p<0.01,***p<0.001, Scale bar = 5 μm).

In agreement with the *in vivo* FRAP data, we also found no significant change in the Proteinase-K-resistant fraction in fresh brain tissue from acutely-treated mice (S2 Fig). Similar to chronic curcumin treatment, we detected an increase in S129-P-α-synuclein in cortical synapses in acutely-treated curcumin mice, compared to DMSO treated controls (DMSO GFP fluorescence = 7659±2327 AU, curcumin GFP fluorescence = 8185±1483 AU, p = 0.4065; DMSO S129-P fluorescence = 12419±690 AU, curcumin S129-P fluorescence = 14931±3302 AU, p<0.001; DMSO S129-P number = 447±143, curcumin S129-P number = 523±238; p = 0.2023; DMSO S129-P area = 0.9±0.1 μm^2^, curcumin S129-P area = 1.1±0.2 μm^2^, p<0.01; DMSO S129-P volume = 0.050±0.013 μm^3^, curcumin S129-P volume = 0.066±0.025 μm^3^, p<0.05; Fig 7A–7C). However, when we looked specifically at Syn-GFP-positive synapses, we detected a significant decrease in S129-P levels after 2 weeks of curcumin treatment, as indicated by a smaller Pearson’s colocalization coefficient between GFP and S129-P (DMSO Pearson’s coefficient = 0.19±0.07, curcumin Pearson’s coefficient = 0.11±0.05, p<0.001; Fig 7D and 7E).

Finally, we detected an average of 11.55±2.51 ng/mL free curcumin in plasma from 15-day curcumin treated mice, which were sacrificed 5 hours after the last curcumin treatment (Table 2). Following B-glucuronidase treatment of plasma, we detected an average of 372.89±104.24 ng/mL curcumin, indicating that 96.6±1.5% of curcumin in the blood is glucuronidated 5 hours after i.p. injection (Table 2). Unlike chronic curcumin treatment, there was no correlation between plasma levels of curcumin and S129-P fluorescence intensity, in individual mice that received acute curcumin treatment (Fig 7F).

## Discussion

We have found that transgenic α-synuclein mice show improvements in gait after chronic treatment with a moderate dose of dietary curcumin. For a 500 ppm curcumin diet, daily consumption rates of ~ 4 g of food would result in 2 mg of curcumin consumed per day over a 6 month period. Despite well-established problems of low bioavailability [16,26], many of curcumin’s cellular mechanisms of action occur in the nM to μM range. Indeed, 0.1–1.0 μM concentrations of curcumin can protect against α-synuclein-induced cell death in tissue culture cells, and curcumin was shown to disaggregate α-synuclein aggregates *in vitro* at concentrations of 0.5–5.0 μM [10,12]. Here we extend these *in vitro* findings to show, for the first time in a genetic synucleinopathy mouse model, that a moderate dose of dietary curcumin is sufficient to improve motor phenotype.

Using LC-MS/MS, we measured mean values of ~4.5 ng/mL free curcumin (range 0–11 ng/mL) and ~12.5 ng/mL total curcumin (range 2–27 ng/mL; free and glucuronidated) in plasma from curcumin diet mice. However, these values are probably lower than peak plasma levels actually attained. Plasma was collected in the early afternoon, leaving at least 6 hours between the end of the dark cycle and thus, likely the last significant food consumption time point. Because the half-life of oral curcumin in mouse plasma is 3.5 hours [19], it is likely that curcumin plasma levels significantly declined due to the lag in collection time. The lag between the last feeding and collection may also contribute to some of the variability between samples. A previous group reported 35 ng/mL curcumin in plasma from mice on a 500 ppm diet for 4 months [3], but these authors did not report timing of the plasma collection relative to the dark cycle or last feeding time point. With acute curcumin dosing, we detected mean values of ~12 ng/mL free curcumin (range 9–13 ng/mL) and ~373 ng/mL total curcumin (range 273–481 ng/mL; free and glucuronidated) in plasma from mice that were treated with 15 mg/kg/day (450 μg) i.p. curcumin for 15 days, and sacrificed 5 hours after the last curcumin treatment. This is also lower than previous reports, which found 127 ng/mL curcumin in plasma 4 hours after 140 μg was injected i.p. into an AD mouse model [3]. These differences could be due to variable metabolism in the different mouse strains used.

In this study, in order to measure curcumin’s molecular effects on α-synuclein, half of each mouse brain was fixed for IHC and the other half processed for synaptosome isolation and subsequent biochemistry; therefore, there was insufficient tissue to attempt to measure brain curcumin levels by LC-MS/MS. Curcumin does cross the blood-brain barrier [3,19], and Begum et al. found 0.47 μg/g (1.28 μM) and 0.74 μg/g (2.01 μM) in mouse brain tissue following chronic dietary and i.p. delivery methods, respectively. These authors showed that with 4 months of chronic dosing with a curcumin diet identical to ours (500 ppm curcumin diet), curcumin was highly enriched in mouse brain tissue, with a brain-to-plasma ratio of 13.4 [3]. With acute dosing, using an i.p. dose of curcumin that is half of the dosage used in our study, they found a brain-to-plasma ratio of 5.85, 4 hours after treatment [3]. These results highlight that curcumin, a lipophilic compound, is more stable in brain than in plasma, and can reach concentrations that are within the range of what has been shown to affect α-synuclein cellular function *in vitro* (see above). Interestingly, we found a strong positive correlation between increased levels of S129-P at individual cortical terminals and plasma levels of curcumin, in individual mice that were on a curcumin diet (Fig 4). This underscores the accumulation and stability of curcumin in lipid-rich brain tissue, particularly with chronic treatment, and lends further support to the brain-specific effects of dietary curcumin in our system. Because curcumin is intrinsically auto-fluorescent, we also attempted to directly measure global curcumin fluorescence or curcumin localization to individual presynaptic terminals using *in vivo* cranial window imaging, but did not detect any significant changes in fluorescence at either 1–3 hours or 7–14 days after acute curcumin treatment.

The Syn-GFP mouse mimics human duplication and triplication mutations to model cellular changes that occur due to 2-3-fold over-expression of α-synuclein. The presence of α-synuclein microaggregates in presynaptic terminals, but not cell bodies, makes this mouse an excellent *in vivo* model of early cellular aggregation of alpha-synuclein, which occurs in many diseases including PD, DLB, and related synucleinopathies [15]. Syn-GFP mice do not show extensive neurodegeneration, severe motor decline, or early mortality rates, compared to more severe mouse models which more strongly overexpress α-synuclein bearing point mutations [14]. Despite this mild phenotype, we were able to measure significant changes in motor behavior due to curcumin diet intervention. Of these gait changes, 3 parameters that are associated with PD-like symptoms were found to be abnormally elevated in control diet mice, and 6 months of curcumin diet intervention was sufficient to bring these parameters back into the normal range. In control diet mice, expression of Syn-GFP increased the variability in both stride length and stance width, which are the same gait parameters reported to increase in a toxin-induced mouse model of PD [21]. It would be interesting to examine curcumin diet intervention in the A53T and E46K α-synuclein point mutation mouse models that show more severe motor dysfunction and early mortality [27,28], at varying time points of disease progression, to test whether curcumin treatment could halt or reverse these more severe neurodegenerative phenotypes.

We measured a global increase in S129-P-α-synuclein in cortical presynaptic terminals in mice that were chronically and acutely treated with curcumin. With chronic curcumin treatment, this increase in phosphorylation was also present at Syn-GFP-positive terminals, while in acutely-treated mice we measured a decrease in S129-P at Syn-GFP-positive terminals. Combined, these data indicate that the effects of curcumin on phosphorylation of human Syn-GFP are differentially modulated over acute and chronic treatment time points, despite there being an overall increase in endogenous mouse S129-P for both acute and chronic curcumin exposure. With acute treatment, where S129-P-α-synuclein is decreased in terminals containing human Syn-GFP but increased in terminals containing endogenous mouse α-synuclein, this could represent an effect of short-term curcumin treatment specifically on human α-synuclein that is reversed with long-term exposure to curcumin. Human and mouse α-synuclein differ by only 7 amino acids and may functionally compensate for each other, but importantly, endogenous mouse protein contains a threonine at the 53 position that is associated with increased aggregation and toxicity in human patients [9]. It has been proposed that curcumin binds to amino acids 89–91 of α-synuclein [13]; thus if threonine-53 present in mouse protein is responsible for the differential effects of curcumin, it may occur through allosteric changes to the binding pocket. It is also possible that curcumin acts to increase mouse α-synuclein presynaptic terminal expression, which could result in an overall increase in S129-P staining. We did not measure mouse α-synuclein by IHC due to the relative lack of a high quality mouse-specific antibody, but we did find that there was no change in human Syn-GFP levels with acute or chronic treatment. It will also be interesting in future studies to determine which kinases and phosphatases are involved in modifying the presynaptic pool of α-synuclein at S129, and to determine if curcumin may be directly modulating activity of these enzymes to produce the changes that we have measured.

How S129-P phosphorylation of α-synuclein affects aggregation and toxicity is currently under debate. Because S129-P-α-synuclein is highly correlated with disease progression and pathology, with up to 90% of α-synuclein in LBs containing this modification, S129-P has long been thought to be a marker of toxic forms of the protein [29,30]. However, recent evidence indicates that S129-P modification targets the protein for degradation via the autophagy pathway, and phosphorylation at this site was found to be neuro-protective both *in vitro* and *in vivo* [31]. We found that mice on a curcumin diet showed improvements in motor behavior as well as a global increase in S129-P in cortical pre-synaptic terminals, which supports the hypothesis that S129-P is protective in our synucleinopathy mouse model. The decrease in S129-P at Syn-GFP synapses after 2 weeks of curcumin treatment did not correlate with any changes in Syn-GFP terminal microaggregates, as measured by *in vivo* FRAP experiments and Proteinase-K digestion, indicating that longer time points of treatment may be needed to measure a direct effect of curcumin on α-synuclein aggregation.

There are a number of possible mechanisms for how curcumin treatment increases S129-P-α-synuclein and improves motor behavior. Curcumin has been shown to increase proteasome activity and heat shock protein expression at low doses (up to 1 μM) *in vitro*, and to induce autophagic clearance of A-beta, which resulted in attenuated cognitive impairment in an AD mouse model [32,33]. Degradation of α-synuclein can occur through both proteasome and autophagy pathways, and S129-P has been shown to directly target α-synuclein for autophagic degradation to provide neuroprotection and motor improvement in another mouse model of PD [31,34]. There is also evidence that phosphomimic of S129 inhibits binding of pore-forming α-synuclein oligomers to membranes, thus conferring protection by blocking this form of toxic oligomers [35]. Thus curcumin’s action to increase α-synuclein phosphorylation may specifically promote its degradation via autophagy or proteasomal pathways, or by blocking the membrane-binding activity of toxic oligomers. Another possible mechanism involves direct disaggregation of α-synuclein by curcumin binding. Curcumin can directly bind to and disaggregate both α-synuclein oligomers and fibrils *in vitro*, but phosphorylation state of the protein was not examined in those experiments [12,13]. Furthermore, the Syn-GFP microaggregates that we have previously characterized in these mice are not higher-order oligomers or fibrils, but instead exist as smaller aggregate species that are easily disaggregated into a single Syn-GFP band by SDS-PAGE analysis, consistent with this mouse being a model for early stages in the aggregate cascade [15]. We found no evidence that acute or chronic curcumin treatment modulates Proteinase K resistance of microaggregates in our mice, however FRAP experiments were only performed on acute curcumin treated mice. To test for a possible direct interaction between curcumin and Syn-GFP aggregates, further biochemical experiments would be necessary to characterize these aggregates, possibly by native western blot analysis or sequential SDS/Triton/Urea extraction. It would also be beneficial to use aggregate-specific antibodies, or antibodies to other post-translational modifications, to examine how curcumin may be modulating these different aggregate forms. For example, there is evidence that α-synuclein aggregates undergo nitration-based oxidative damage [36]. Curcumin is known to be a potent anti-oxidant and may be modulating α-synuclein, and ultimately motor behavior, by decreasing reactive oxygen species [11]. While further studies are needed to better understand the molecular mechanisms underlying chronic curcumin diet intervention in synucleinopathy mouse models, our data demonstrate improvements in motor behavior, providing strong support for curcumin therapy as the subject of further pre-clinical studies.

## Supporting Information

S1 Fig No curcumin or S129-P-α-synuclein staining in control human brain tissue. (A) Individual fluorescence spectra of curcumin, Alexa-647 secondary antibody, and two autofluorescent components, as defined by spectral imaging and linear unmixing in DLB tissue. Autofluorescence components (B, C) are present in control human brain tissue, but no curcumin-positive (D) or S129-P-α-synuclein-positive (E) structures were found (F, merge; scale bar = 10 μm).
(TIF)

S2 Fig No change in Proteinase-K-resistant aggregates in acutely treated curcumin mice. (A) Western blot detection of Syn-GFP microaggregates, following Proteinase-K digestion of synaptosome and cytosolic protein fractions from mice treated with DMSO control or 15 mg/kg/day curcumin for 2 weeks. (B) Quantification of Syn-GFP band intensity shows no change in the resistant fraction between control and curcumin diet mice.
(TIF)
