# Supplementary figures and images for: Curcumin Treatment Improves Motor Behavior in α-Synuclein Transgenic Mice
Source: PLoS One. 2015 Jun 2;10(6):e0128510. doi: 10.1371/journal.pone.0128510 (PMC4452784; doi:10.1371/journal.pone.0128510)

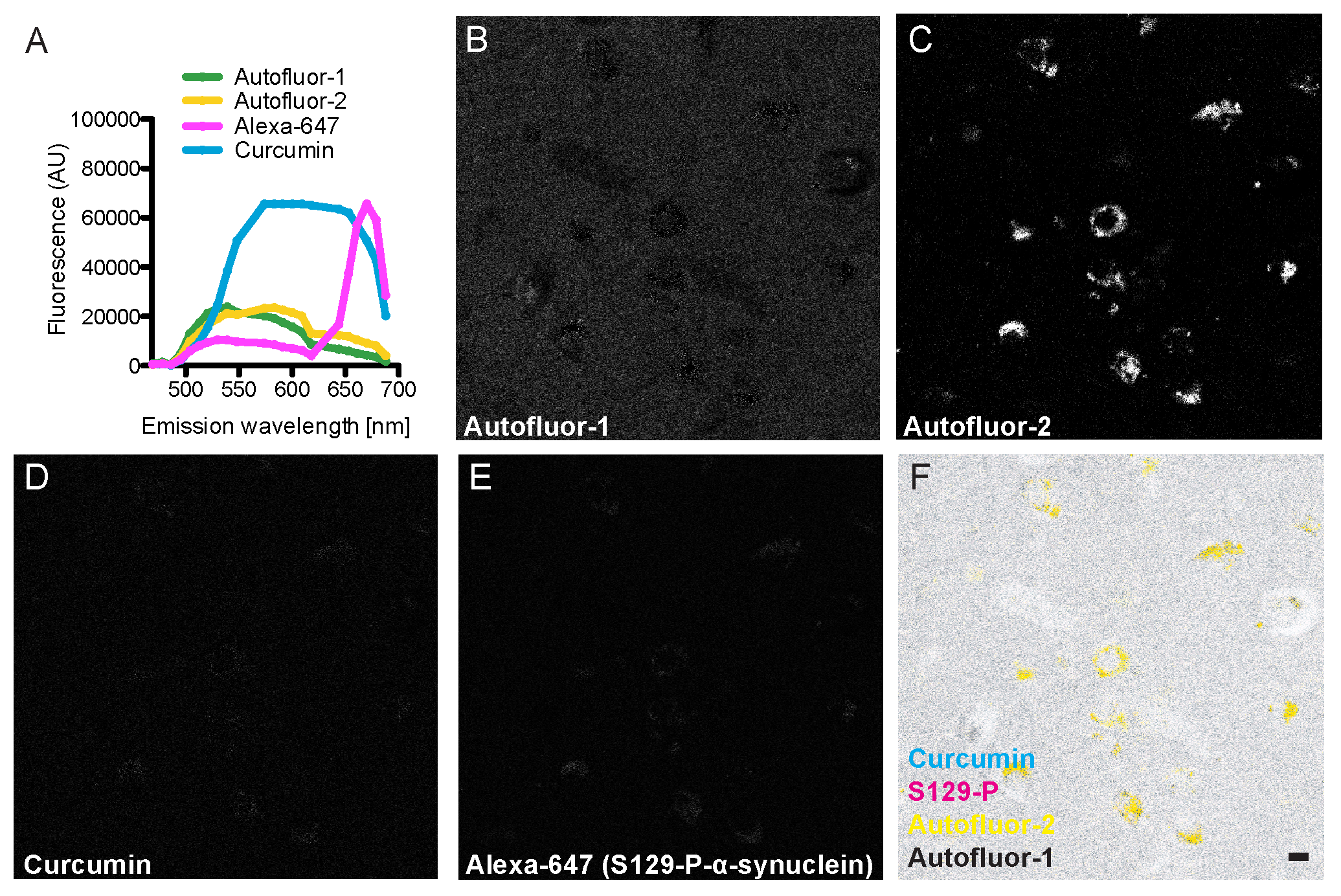

Supplement: S1 Fig — (A) Individual fluorescence spectra of curcumin, Alexa-647 secondary antibody, and two autofluorescent components, as defined by spectral imaging and linear unmixing in DLB tissue. Autofluorescence components (B, C) are present in control human brain tissue, but no curcumin-positive (D) or S129-P-α-synuclein-positive (E) structures were found (F, merge; scale bar = 10 μm). (TIF) [file pone.0128510.s001.tif]

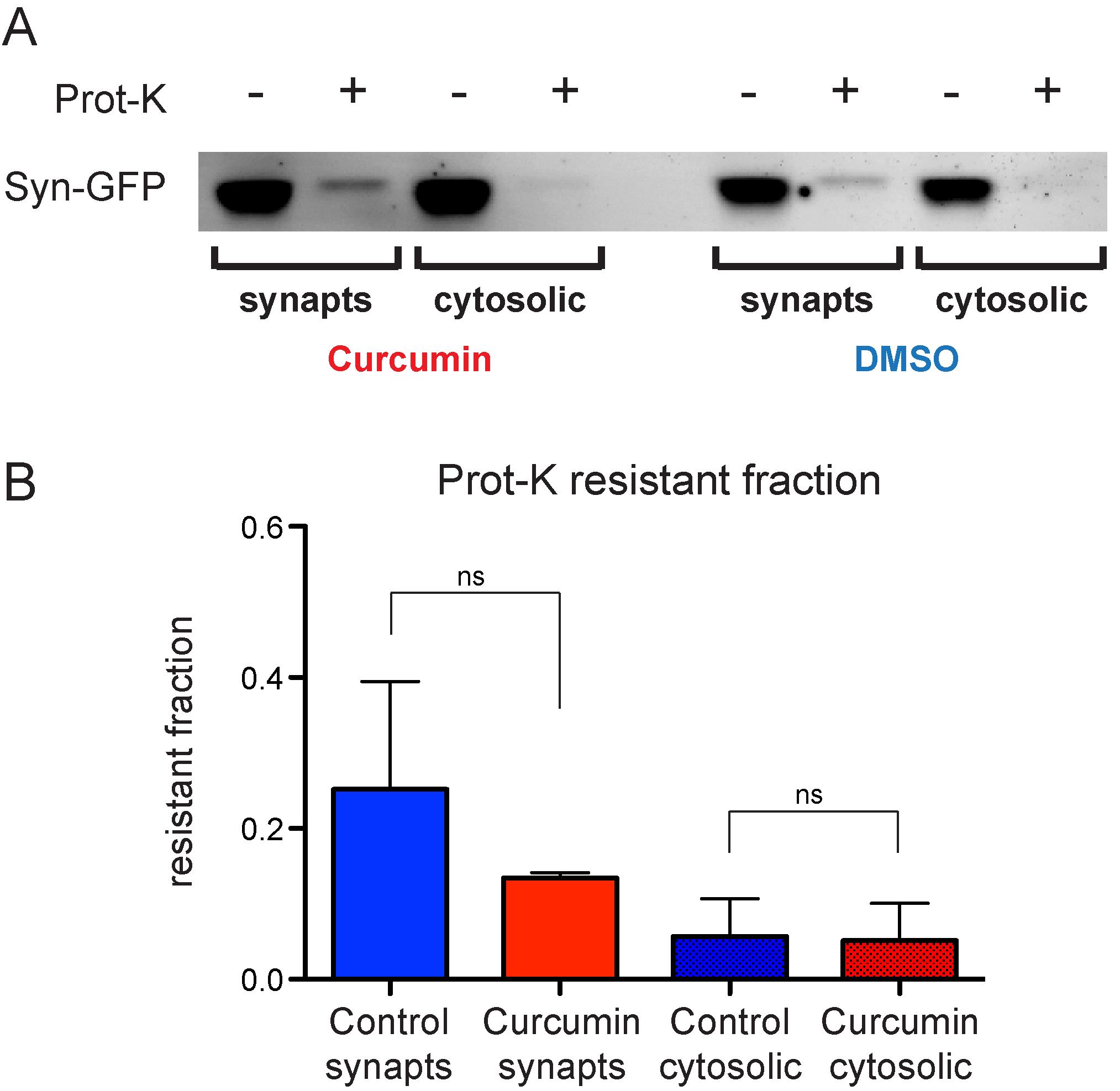

Supplement: S2 Fig — (A) Western blot detection of Syn-GFP microaggregates, following Proteinase-K digestion of synaptosome and cytosolic protein fractions from mice treated with DMSO control or 15 mg/kg/day curcumin for 2 weeks. (B) Quantification of Syn-GFP band intensity shows no change in the resistant fraction between control and curcumin diet mice. (TIF) [file pone.0128510.s002.tif]
